# Comparison of temporalis fascia muscle and full-thickness cartilage grafts in type 1 pediatric tympanoplasties^☆^

**DOI:** 10.1016/j.bjorl.2015.12.009

**Published:** 2016-03-28

**Authors:** Yakup Yegin, Mustafa Çelik, Arzu Karaman Koç, Levent Küfeciler, Mustafa Suphi Elbistanlı, Fatma Tülin Kayhan

**Affiliations:** Bakırköy Dr. Sadi Konuk Training and Research Hospital, Department of Otorhinolaryngology – Head and Neck Surgery, Istanbul, Turkey

**Keywords:** Child, Fascia, Hearing, Thickness, Tragal cartilage, Tympanoplasty, Criança, Fáscia, Audição, Espessura, Cartilagem tragal, Timpanoplastia

## Abstract

**Introduction:**

Various graft materials have been used to close tympanic membrane perforations. In the literature, there are few studies in pediatric populations comparing different graft materials. To our knowledge, there is no reported study that measured the thickness of the tragal cartilage in pediatric tympanoplasties. The tragal cartilage is not of uniform thickness in every patient.

**Objective:**

To compare anatomical and functional outcomes of temporalis fascia muscle and full-thickness tragal cartilage in type 1 pediatric tympanoplasties.

**Methods:**

In total, 78 patients (38 males, 40 females; average age 10.02 ± 1.98 years; range, 7–18 years) who underwent type 1 tympanoplasties in our clinic were included. Demographics, anatomical, and functional outcomes were collected. Temporalis fascia muscle and tragal cartilage were used as graft materials. Tragal cartilage was used without thinning, and the thickness of tragal cartilage was measured using a micrometer. Anatomical and functional outcomes of cartilage and fascia were compared. Audiometric results comparing the cartilage and fascia groups were conducted at 6 months, and we continued to follow the patients to 1 year after surgery. An intact graft and an air-bone gap ≤ 20 dB were regarded as a surgical success. Results with a p-value < 0.05 were considered statistically significant.

**Results:**

The graft success rate was 92.1% for the cartilage group compared with 65.0% for the temporal fascia group. In the fascia group, the preoperative air-bone gap was 33.68 ± 11.44 dB and postoperative air-bone gap was 24.25 ± 12.68 dB. In the cartilage group, the preoperative air-bone gap was 35.68 ± 12.94 dB and postoperative air-bone gap was 26.11 ± 12.87 dB. The anatomical success rate in the cartilage group was significantly better than that for the fascia group (*p* < 0.01). There was no statistically significant difference in functional outcomes between the fascia and cartilage groups (*p* > 0.05). The average thickness of tragal cartilage in the pediatric population was 0.693 ± 0.094 mm in males and 0.687 ± 0.058 mm in females.

**Conclusions:**

Our data suggest that the anatomical success rate for a cartilage tympanoplasty was higher than for a fascia tympanoplasty. Functional results with cartilage were not different than with fascia, even though we did not thin the tragal cartilage. However, further studies should focus on the interaction between the thickness of the tragal cartilage and the tympanoplasty success rate.

## Resumo

**Introdução:**

Vários materiais de enxerto foram usados para o fechamento da perfuração da membrana timpânica. Há poucos estudos na literatura que comparam diferentes materiais de enxerto em populações pediátricas. De acordo com nossa pesquisa, não há estudo que tenha medido a espessura da cartilagem tragal em timpanoplastia pediátrica. A espessura da cartilagem tragal não é uniforme em todos os pacientes.

**Objetivo:**

Comparar os resultados anatômicos e funcionais da fáscia do músculo temporal e da cartilagem tragal com espessura total em timpanoplastias tipo 1 em crianças.

**Método:**

No total, 78 pacientes (38 do sexo masculino, 40 do sexo feminino; média de idade de 10,02 ± 1,98 anos; variação: 7-18 anos) submetidos a timpanoplastia tipo 1 em nossa clínica foram incluídos. Os resultados demográficos, anatômicos e funcionais foram registrados. A fáscia do músculo temporal e a cartilagem tragal foram usadas como materiais de enxerto. A cartilagem tragal foi usada sem fresagem e a sua espessura foi medida com um micrômetro. Os resultados anatômicos e funcionais da cartilagem e da fáscia foram comparados. Os resultados audiométricos comparando os grupos (cartilagem e fáscia) foram avaliados em 6 meses, e o acompanhamento dos pacientes prosseguiu até 1 ano após a cirurgia. Enxerto intacto e um intervalo aéreo-ósseo (*gap*) ≤ 20 dB foram considerados como sucesso cirúrgico. Os resultados com um valor *p* < 0,05 foram considerados estatisticamente significantes.

**Resultados:**

A taxa de sucesso do enxerto foi de 92,1% para o grupo cartilagem e de 65,0% para o grupo fáscia temporal. No grupo fáscia, o *gap* no pré-operatório foi 33,68 ± 11,44 dB, e 24,25 ± 12,68 dB no pós-operatório. No grupo cartilagem, o *gap* no pré-operatório foi 35,68 ± 12,94 dB, e 26,11 ± 12,87 no pós-operatório. A taxa de sucesso anatômico no grupo cartilagem foi significantemente melhor que a do grupo fáscia (*p* < 0,01). Não houve diferença estatisticamente significante nos resultados funcionais entre os grupos fáscia e cartilagem (*p* > 0,05). A espessura média da cartilagem tragal na população pediátrica foi 0,693 ± 0,094 mm em meninos e 0,687 ± 0,058 mm em meninas.

**Conclusões:**

Nossos dados sugerem que a taxa de sucesso anatômico para uma timpanoplastia com enxerto de cartilagem seja maior que a de uma timpanoplastia com enxerto de fáscia. Os resultados funcionais com cartilagem não foram diferentes daqueles com fáscia, embora não tenhamos fresado a cartilagem tragal. Porém, novos estudos devem concentrar-se na relação entre a espessura da cartilagem tragal e a taxa de sucesso da timpanoplastia.

## Introduction

A tympanoplasty is a surgical procedure to close a tympanic membrane perforation and reconstruct the tympanic membrane and hearing, commonly after chronic otitis media and trauma. In the literature, reconstructing a tympanic membrane perforation was first described by Berthold^1^ in 1878, and the term ‘tympanoplasty’ was introduced in 1952 by Zollner^2^ and Wullstein.^3^ A tympanic membrane perforation is frequently present as a sequela of middle ear infections, trauma, and iatrogenic causes.

A tympanoplasty is fundamentally a tissue transference procedure. Various graft materials have been used to close tympanic membrane perforations. These graft materials include temporalis fascia muscle, cartilage, pericondrium, periostia, dura mater, vein tissue, fat, and skin.^4^, ^5^ Many previous studies have compared anatomical and functional outcomes of various graft materials. Anatomical success is typically defined as an intact graft and dry ear on the operated side. The functional definition of a successful graft is an air-bone gap (ABG) ≤20 dB. Today, the most commonly used graft material is temporalis fascia muscle.

Recently, a cartilage graft has begun to be used to reconstruct perforations of the tympanic membrane. In 1963, a cartilage graft for reconstructing a tympanic membrane perforation was introduced by Salen and Jansen.^4^ Cartilage tympanoplasty methods were divided into six main groups by De Seta et al.^5^ Cartilage is resistant to retraction and infection and preserves its viability and shape for a long time in the presence of middle ear pathologies. In the literature, there are few studies in pediatric populations comparing different graft materials; the reported studies were performed mostly in adults.

In the present study, we compared anatomical and functional outcomes of full-thickness tragal cartilage and fascia in type 1 pediatric tympanoplasties in patients with low middle ear risk index scores who had similar middle ear pathologies. We also aimed to determine the average thickness of tragal cartilage in the pediatric population.

## Methods

A retrospective review of data collected from January 2013 to September 2014 was performed at our hospital, at the Department of Otolaryngology, Head and Neck Surgery. In total, 78 children who underwent type 1 tympanoplasties with low middle ear risk index scores were included in this study. Patients with ossicular chain defects, cholesteatoma, tympanosclerosis, atelectasia, a history of previous ear surgery, irregular follow-up, and no written informed consent were excluded. All patients were followed for at least six months after surgery at our clinic. All patients had pure tone audiograms preoperatively. All patients had surgery under general anesthesia with a retroauricular approach with the overlay-underlay technique.

The age, gender, dimensions of the perforation, the side of the operated ear, the types of graft materials, middle ear pathologies, pre- and postoperative audiological outcomes, the status of the graft in the postoperative period, and follow-up period were recorded for all patients. Middle ear pathologies of all patients were evaluated using the Middle Ear Risk Index (MERI) scoring system, developed by Kartush.^6^ We standardized our patients with this scoring system and used it to prevent selection bias between the groups. Patients with MERI scores >3 were excluded. Tympanic membrane perforations were classified as medium (<50%), subtotal (>50%), and total (100%).

All operations were performed by one of the surgeons in our department according to established principles of ear surgery. The patients were randomly allocated to surgery using temporalis fascia muscle or tragal cartilage grafts by the surgeons. The choice of graft type to be used in the tympanoplasty was explained to all patients. The patients included in the study were divided into two groups according to the graft material used. In the fascia group, temporalis fascia muscle was used as the graft material. The temporalis fascia muscle was harvested with a retroauricular incision. The tympanomeatal flap was prepared after incisions in the 6 and 12 o’clock directions. After it was shaped, according to the size of the perforation, it was placed over the malleus and under the anterior annulus with an overlay-underlay technique. Gelfoam was used to support the graft medially and laterally. The tympanomeatal flap was then repositioned, and a meatal pack was added. The incision was sutured, and a mastoid bandage was used.

**Figure 1:**
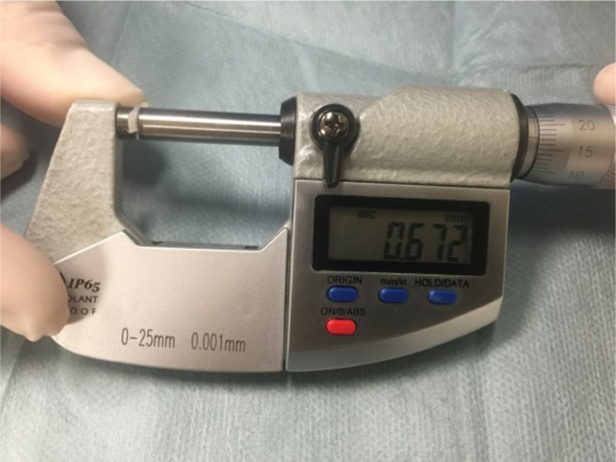
Measurement of the thickness of tragal cartilage using a micrometer.

In the cartilage group, tragal cartilage was used as the graft material without thinning. To avoid a visible scar, a skin incision was made 1 mm inside the ear canal. The outer edge of the tragus protected against a cosmetic deformity. The tragal cartilage was harvested together with perichondrium on both sides. An inferior cut was made as low as possible to gain the whole tragal cartilage. The perichondrium was peeled at the convex side, and a triangular piece of cartilage was removed at the malleus level for a better fitting graft. According to Tos,^7^ this technique is categorized as a cartilage-perichondrium composite island technique. The surgical procedure was otherwise similar to the first group. The thickness of the tragal cartilage was measured with a micrometer and recorded intraoperatively. The measurements of thickness were performed by the same surgeon. All measurements were repeated by a second surgeon to reduce inter-observer variation. Measurements were made of the superior, middle, and inferior part of the tragal cartilage (Fig. 1).

The meatal pack and mastoid bandage were removed after 72 h. All patients were examined post-operatively, and at the first, second, and third weeks following. In the first postoperative week, the sutures were removed, and in the third postoperative week, the external ear canal was cleaned of Gelfoam particles. The patients were then examined at monthly intervals during the postoperative first year. The status of the tympanic membrane was recorded at the postoperative first, third, and sixth months. Audiological evaluations were made at the postoperative sixth month.

In this study, anatomical success was defined as an intact graft without perforation, retraction, or lateralization, and a dry ear on the operated side. Functional success of the operation was defined as an air-bone gap (ABG) ≤20 dB.

All patients were informed about this study and written informed consent was obtained from the patients who participated. The study protocol was approved by the hospital's local ethics committee (ethical committee number 2015/162).

### Statistical analysis

The Number Cruncher Statistical System (NCSS) 2007 software (UT, USA) was used for statistical analyses. Data were evaluated using descriptive statistical methods (mean, standard deviation, median, and interquartile range). The significance of intergroup differences was analyzed using Student's *t*-test, and the significance of the medians was analyzed with the Mann–Whitney *U*-test. A paired *t*-test was performed to test differences between preoperative and postoperative anatomical and functional outcomes. Qualitative comparisons of data were performed using the *χ*^2^ test and the Fisher–Freeman–Halton test. Results with a *p*-value < 0.05 were considered statistically significant.

## Results

In total, 78 patients were included, 40 (61.5%) females and 38 (38.5%) males. Their average age was 10.02 ± 1.98 years (range: 7–18). There were 40 patients in the fascia group and 38 in the cartilage group. The mean follow-up period was 16.1 ± 9.9 months (range: 12–33). The characteristics of the groups are summarized in Table 1. Systemic diseases were determined in 17% of all patients. Tympanoplasties were performed in 41 (52.5%) of right ears and 37 (47.5%) of left ears. The age, gender, side of surgery, tympanic membrane perforation size and MERI scores were not significantly different between the groups (*p* > 0.05). In the fascia group, the pre- and postoperative mean ABGs were 33.68 ± 11.44 dB and 24.25 ± 12.68 dB, respectively; the postoperative gain was 9.42 ± 8.91 dB in the fascia group. In the cartilage group, pre- and postoperative mean ABGs were 35.68 ± 12.94 dB and 26.113 ± 12.87 dB, respectively; the postoperative gain was 12.57 ± 9.34 dB in the cartilage group (Table 2).

**Table 1:** Subject data in the fascia and cartilage groups.

Variables	Fascia (*n* = 40)	Cartilage (*n* = 38)	*p*
*Age (years)*	10.17 ± 1.97	10.95 ± 1.88	0.604^a^
*Gender*			
Females	24 (60.0)	24 (63.2)	0.957^c^
Males	16 (40.0)	14 (36.8)	

*The side operated*			
Right	24 (60.0)	17 (44.7)	0.262^c^
Left	16 (40.0)	21 (55.3)	
MERI	2.30 ± 1.64 (1)	2.01 ± 1.31 (1)	0.295^b^

*Graft intact*			
Perfore	14 (35.0)	3 (7.9)	0.009^c^^,^^d^
Intact	26 (65.0)	35 (92.1)	

*Perforation size*			
Medium (25–50)	15 (37.5)	18 (47.4)	0.274
Total (100%)	0	1 (2.6)	0.778
Subtotal (>50)	25 (62.5)	19 (50.0)	0.342

MERI, Middle Ear Risk Index.

a Independent samples test.

b Yates continuity correction.

c Fisher's exact test.

d *p* < 0.01.

**Table 2:** Comparison of air-bone gap and the hearing gains between the two groups pre- and postoperatively.

Air bone gap	Preoperative ABG (dB)	Postoperative (dB)	*p*^a^	Gain (median)	*p*^b^
*Graft type; mean* ± *SD*					
Fascia	33.68 ± 11.44	24.25 ± 12.68	0.001^c^	9.42 ± 8.91	0.968
Cartilage	35.68 ± 12.94	26.113 ± 12.87	0.001^c^	12.57 ± 9.34	

a Paired samples test. Comparison ABG between two groups pre- and postoperatively.

b Mann–Whitney *U* test. Comparison between two groups in terms of gain.

c *p* < 0.01.

The functional success rate in the cartilage group was higher than in the fascia group but no statistically significant difference in functional success was observed (*p* > 0.05). The graft success rate was 92.1% in the cartilage group and 65.0% in the fascia group; the graft success rate in the cartilage group was significantly higher than in the fascia group (*p* < 0.001; Table 3).

**Table 3:** Comparison of success rates between the groups.

	Cartilage group (*n* = 38)	Fascia group (*n* = 40)	*p*^a^
Graft success	92.1% (35)	65.0% (26)	0.009^b^
Hearing success (ABG ≤ 20 dB)	76.3% (29)	75.0% (30)	1.000

a Fisher's exact test.

b *p* < 0.01.

The total average thickness of the tragal cartilage was 0.693 ± 0.094 mm in males and 0.687 ± 0.058 mm in females (Table 4). The average thickness of the tragal cartilage was not significantly different between males and females (*p* > 0.05).

**Table 4:** Average thickness of tragal cartilage with regard to gender.

Gender	Patients (*n*)	Mean thickness ± SD (mm)
Males	30	0.693 ± 0.094
Females	48	0.687 ± 0.058

SD, standard deviation.

## Discussion

There have been few studies of pediatric tympanoplasties comparing different materials and measuring tragal cartilage. Moreover, there is no consensus on the selection of graft materials for tympanoplasties; it depends entirely on surgeon experience and preferences. There is also no consensus on the best surgical age, surgical procedure, graft materials, or postoperative care modalities in pediatric tympanoplasties. This lack of data hinders reaching consensus on controversial issues. Repeated upper respiratory tract infections, shorter and unpredictable function of the eustachian tube (ET), and difficulties in postoperative care in children affect the success rates of pediatric tympanoplasties. In the study of Dinç et al.,^8^ it was shown that the more horizontal angle and shorter length of the ET influenced the development of chronic otitis media. In children, the ET is more horizontal and shorter than in adults.^9^ To our knowledge, there is no reported study that focused on the interaction between the angle and length of the ET and the success rate of pediatric tympanoplasties. The success rate of tympanoplasties without complete maturation of the ET seems to be lower. In the literature, the maturation of the ET is complete at approximately 6 years of age.^9^, ^10^ However, some authors suggest performing a tympanoplasty at an earlier age, considering the complications and sequelae of chronic otitis media.^11^ In the retrospective study of Duval et al.,^12^ it was shown that children younger than 4 years had the worst outcomes in pediatric tympanoplasties. Variable outcomes have been reported in studies investigating the effects of gender and success rates in tympanoplasties. In the study of Emir et al.,^13^ a good correlation was shown between males and success rate, in contrast to the Vartiainen et al.^14^ study results.

In the literature, there is no consensus on graft materials for pediatric tympanoplasties. Generally, some previous studies compared the anatomical and functional success rates of cartilage and fascia grafts. Demirci et al.^15^ reported the anatomical success rate was 92% in the cartilage group and 82.9% in the fascia group, with no significant difference in functional success between the groups. Likewise, Dornhoffer et al.^16^ reported no significant difference in functional success between the groups. Özbek et al.^17^ reported that the anatomical success rate was 100% in palisade cartilage tympanoplasties and 72% in the fascia group and that the anatomical success was significantly statistically higher in the cartilage group than in the fascia group. However, they reported no significant difference in functional success between the groups. Similarly, Albirmawy et al.^18^ found that anatomical success was significantly higher in the cartilage-perichondrium composite ring group than in the temporalis fascia muscle group in pediatric tympanoplasties. In another pediatric study, Couloigner et al.^19^ reported no significant difference in graft success or hearing gain between inlay butterfly cartilage and fascia tympanoplasties. Tragal cartilage is a fibroelastic cartilage and being composed of collagen type II is similar nature of the tympanic membrane; temporalis muscle fascia consists primarily of collagen type I. Collagen type II has higher tensile strength than other types.^20^ The superiority of cartilage with regard to tympanic membrane closure is thought to be derived from its rigidity, a characteristic that seems especially important in ears with eustachian tube dysfunction. In the present study, the success rate of the temporalis fascia group was significantly lower than might be expected from a recently published meta-analysis. The results of the present study show no difference in terms of audiological outcomes between the two techniques, but higher rate of reperforation (35% vs. 8%) occurred in fascia groups. Confounding variables that were addressed include the surgical approach used, experience and skills of the surgeons, and the sizes of the tympanic membrane perforations. Regarding higher rate of reperforation in fascia group, it is notable that size and location of perforation differed between studies, and that some studies did not describe either of these perforation characteristics. Small perforations have a comparatively good preoperative hearing and are easier to close. In the present study, tympanic membrane perforation was mainly subtotal perforations; there might be heterogeneity between recently published meta-analysis. Secondly, there is heterogeneity between the present study and published studies with regard to length of follow-up. Thirdly, experience of surgeon can affect the success rate of tympanoplaties. The success rate of tympanoplasty is higher in more expert surgeons compared to inexperienced surgeons. In our clinic, the fascia tympanoplasties were generally performed by resident surgeons and the low success rate of the fascia group may be attributed to surgeon experiences. Yilmaz et al.^21^ reported that cartilage type 1 tympanoplasties were effective in both children and adult patients.

To our knowledge, there is no reported study that measured the thickness of the tragal cartilage in pediatric tympanoplasties. The tragal cartilage is not of uniform thickness in every patient. Also, we do not know the effects of race, age, or gender in the changing thickness of the cartilage. Khan et al.^22^ found the total average thickness of the tragal cartilage was 1.228 ± 0.204 mm in males and 1.090 ± 0.162 mm in females in an Indian population. In our study, the thickness of the tragal cartilage was measured with a micrometer and recorded intraoperatively. Tragal cartilage was used without thinning; the average thickness of the tragal cartilage was 0.693 ± 0.094 mm in males and 0.687 ± 0.058 mm in females in the present study population. The present study provides the first report of measured thickness of tragal cartilage in pediatric tympanoplasties.

The success rate of tympanoplasties decreases with time. The success rates of tympanoplasties with long-term follow-up were lower than with short-term follow-up.^15^, ^23^ In the present study, all patients were followed for at least one year. Longer follow-up is essential after tympanoplasty operations, because repaired membranes often reperforate, especially when the initial perforation is subtotal or total.^24^ Our follow-up period was sufficiently long to see some reperforations.

There is no consensus on the selection of graft materials for tympanoplasties with similar MERI scores. Callioğlu et al.^23^ reported there was no significant difference in ABG closure in tympanoplasties with low MERI scores. In the present study, there was no difference in MERI scores between the fascia and cartilage groups. Limitations of this study included a study design with multiples surgeons, the sample size and a lack of randomization. The main limitation of this study is participation of multiple surgeons and the lack of randomization. If the study had been performed by an unique surgeon and the study design were a randomized study, it would perhaps be more valuable.

## Conclusions

In conclusion, the anatomical success rate was higher with cartilage grafts. Further studies with larger numbers of patients are needed to compare the anatomical and functional outcomes of various cartilage types and differing thickness of cartilage grafts in pediatric tympanoplasties.

## Funding

The authors declare that this study did not receive financial support.

## Conflicts of interest

The authors declare no conflicts of interest.
